# Emotional experiences of medical students during cadaver dissection and the role of memorial ceremonies: a qualitative study

**DOI:** 10.1186/s12909-018-1358-0

**Published:** 2018-11-12

**Authors:** Hyung-Joo Chang, Hyun Jung Kim, Im Joo Rhyu, Young-Mee Lee, Chang-Sub Uhm

**Affiliations:** 1 Department of Medical Education, Korea University College of Medicine, 73 Inchon-ro, Seongbuk-gu, Seoul, 02841 South Korea; 2 Graduate School of Medical Science and Engineering, Korea Advanced Institute of Science and Technology, 291 Daehak-ro, Yuseong-gu, Daejeon, 34141 South Korea; 3 Department of Anatomy, Korea University College of Medicine, 73 Inchon-ro, Seongbuk-gu, Seoul, 02841 South Korea

**Keywords:** Gross anatomy education, Professionalism education, Medical students, Memorial ceremonies, Cadaver donors, Emotional experiences

## Abstract

**Background:**

It has been well reported that the emotional experiences of medical students in the gross anatomy laboratory could have significant impacts on their professional identity formation. This qualitative study aimed to investigate students’ emotions towards cadaver dissection and the educational role of memorial ceremonies.

**Methods:**

A total of 37 students from eight teams were recruited in the team-based dissection course during two consecutive academic years (2016 and 2017) at one medical school. In focus group interviews, students were encouraged to express and discuss their emotions regarding cadaver dissection and memorial ceremonies.

**Results:**

The participants described their apprehension and anxiety during their first encounter with cadavers that diminished through gradual exposure. Unfortunately, their positive emotions such as gratitude and responsibility also tended to decline under the pressure of excessive workloads and frequent examinations. Memorial ceremonies, including not only large-scale events but also daily rituals, had educational effects that they prevented the decline of students’ responsibility and respect during the dissection course.

**Conclusion:**

Educators should assist medical students in overcoming their initial distress and maintaining respectful attitudes throughout the dissection course. Memorial ceremonies can be effective educational tools for fostering appropriate attitudes and ethical practice in the gross anatomy laboratory.

**Electronic supplementary material:**

The online version of this article (10.1186/s12909-018-1358-0) contains supplementary material, which is available to authorized users.

## Background

The gross anatomy laboratory has a significant educational impact on medical students not only in terms of consolidating their knowledge and skills on human bodies, but also internalizing their attitude towards death and caring [1]. Since students’ emotional experiences and self-reflection during the dissection course are known to play an important role in their professional identity formation [2, 3], medical academics have attempted to investigate their emotional responses to cadaver dissection and sublimate those emotions into positive educational experiences [4, 5].

Several studies report that medical students experience negative emotional or physical reactions as they begin cadaver dissection such as shock, anxiety, apprehension, nausea, or dizziness, but overcome these sensations rapidly, perceiving dissection as a challenging task [6–10]. Although previous studies assess students’ emotions through validated scales, they predominantly focus on primary emotional reactions that are typically immediate and transient. A deeper understanding of the complex emotions of medical students and their variations during cadaver dissection is required to promote medical professionalism in undergraduate education.

Many medical schools worldwide conduct memorial ceremonies of gratitude towards cadaver donors in order to promote humanistic values such as respect, altruism, and responsibility among students [11–13]. However, few studies have attempted to explore students’ emotional and cognitive responses to memorial ceremonies or their educational influence. Especially, qualitative studies to deeply understand the educational role of memorial ceremonies in the gross anatomy laboratory have rarely been reported.

Our school, the Korea University College of Medicine has a strong tradition of the memorial ceremonies as it organizes a 12-week cadaver dissection course for first-year students. There are two annual ceremonies at the commencement and the middle of the course. During the ceremony on the first day (dae-myun-sik), anatomy professors provide lectures regarding professional attitudes when dealing with human cadavers. Subsequently, all the students participate in shaving the hair from donors’ bodies after a short tribute while facing the dissection tables. The annual ceremony with donors’ families (gam-eun-je) is a half-day event where students, professors, and family members pay tribute to the donors together before the memorial tower on our campus. Besides these major events, students offer brief silent tributes daily at both the beginning and the end of dissections. The leader of each dissection team directs the rituals without any intervention from teachers. In addition, every dissection table has a signboard that provides a brief personal history and photograph of the donor and his/her will for the students.

### Aim

This qualitative study aims to investigate the diverse emotional experiences of medical students during cadaver dissection and the reasons behind those responses. Also, this study intends to identify the educational role of memorial ceremonies for cadaver donors.

## Methods

### Study design and sample

A qualitative, exploratory study was conducted by employing focus group interviews with medical students during two consecutive school years (2016 and 2017) at the Korea University College of Medicine, Seoul, South Korea. At this school, all first-year medical students perform cadaver dissections in the gross anatomy laboratory for 12 weeks (from March to May). The students are randomly organized into dissection teams of four or five, and each team dissects one cadaver throughout the curriculum. The 12-week schedule of cadaver dissection is presented in Appendix 1 (see Additional file 1). The dissection course runs in parallel with the theoretical anatomy class for letting the students instantly apply their knowledge to practice. During the dissection, there is a checklist of anatomical structures that should be identified each day. Appendix 2 shows an example of the checklists (see Additional file 2).

In 2016, at the commencement of the course, the authors provided all the students with complete information regarding the study. Hence, four teams among the 20 dissection teams altogether, voluntarily participated in the study. Since each team consisted of four or five students, a total of 18 students were included in the focus group interviews. In 2017, the authors similarly recruited 19 students in four teams. The average age of the 37 subjects was 21.9 (*SD* = 0.82, range = 20 to 28), of whom 23 were male.

### Data collection

The focus group interviews were conducted in April immediately following the annual ceremony with donors’ families by the original dissection team in order to stimulate discussion among students who shared similar experiences. One of the authors (HJC) who did not participate in anatomy education at all, conducted all the interviews during both years to ensure students were comfortable in revealing their emotions and perspectives regarding their learning experiences. Anatomy teachers were excluded from the interviews, even though they had deeper understanding of the dissection process, because the students might feel some pressure to express positive perspectives on anatomy education.

Table 1 presents the semi-structured interview protocol employed in the series of focus group interviews. The interview questions were developed through the preliminary surveys. In 2016, we asked all students what they felt in memorial ceremonies with the short open-ended questionnaire after the two annual events. After reviewing the students’ answers, we developed the question items to investigate their complex emotions towards cadaver dissection and memorial ceremonies.

**Table 1:** Semi-structured interview questions for the focus group interviews

1. Emotional experiences during cadaver dissection	
How did you feel about cadaver dissection before entering the course?	
How do you feel about it now?	
(If there is a change) What made you feel differently?	
How do you feel about your cadaver donor?	
Can you empathize with his/her intention or will regarding the donation?	
2. Educational impact of memorial ceremonies	
How do you feel about the memorial ceremonies for cadaver donors?	
How did these ceremonies influence your emotion or attitude?	
Do you consider these ceremonies indispensable in the gross anatomy laboratory?	

During the interviews, the interviewer minimized intervention after asking questions to encourage active discussion among group members. The interviews ranged from 65 to 84 min, with a mean duration of 73 min. All interviews were audio recorded, and transcribed by an independent transcriptionist who was not a member of our research team to secure unbiased transcripts for further analysis. All data were protected confidentially, using codes instead of identifiers that were available only to the research team.

### Data analysis

Following a literature review on qualitative methodology [14–16], the authors conducted a modified thematic analysis based on grounded theory. The joint research team, comprising experts in medical education and anatomy, analyzed the data in several steps. First, the five researchers on the team, including the interviewer, read each transcript line-by-line and coded it independently. Through open coding, we extracted word descriptors that represented students’ emotional responses to the gross anatomy laboratory, and categorized them. Also, we extracted text descriptors that represented the reasons why they felt a certain emotion. Second, we shared code structures after analyzing every transcript, and adjusted the differences to reach an agreement on a thematic framework. Last, we organized the final categories of the students’ emotions in the gross anatomy laboratory and the reasons for those emotions.

At the beginning of the analysis, we categorized the descriptors into: (1) the emotional experiences in cadaver dissection, (2) the emotional responses to cadaver donors, (3) the emotional responses to memorial ceremonies, and (4) the learning experiences in memorial ceremonies. During the axial coding process, however, we reclassified the descriptors by integrating overlapped contents and modifying the category names to clearly represent the themes. As a results, the categories were determined as: (1) the emotional experiences during cadaver dissection, and (2) the emotional impact of memorial ceremonies.

All the interviews and analysis were done in Korean. The quoted remarks were translated into English for this manuscript, which were cross-checked by a third party who is bilingual in Korean and English.

## Results

### Emotional experiences during cadaver dissection

The interviewees agreed that cadaver dissection was a unique and intense experience that stimulated various emotions. Almost all students mentioned a sense of responsibility and purpose when entering the dissection room. They expressed their gratitude for the opportunity to perform invasive procedures on human bodies. One student remarked:

“The gross anatomy laboratory is the first formal course where we can feel we are medical students. We have the opportunity to demonstrate our theoretical knowledge by operating real human bodies. I think we really have to be grateful for this honor and should do our best to utilize it.”

Most of the medical students experienced rather personal and intimate emotions regarding their cadavers. They seemed to well recognize donors’ intention and will, and expressed gratitude and respect for their altruism. One participant described her relationship with her cadaver donor as follows:

“I consider Mr. OOO (the donor’s name) my first patient. He wanted me to become a competent and compassionate doctor through these dissection experiences. So I feel a similar responsibility and pressure as physicians who treat living patients. I really respect his donation, especially considering our Confucian tradition that has a strong aversion to dissection.”

While most of the students mentioned their responsibility and respect in the gross anatomy laboratory, they also admitted that these positive emotions gradually decreased during the last two months of the dissection course. They indicated that the excessive workload, frequent examinations, and dissection procedures focusing on specific parts of the body contributed to their emotional numbness. One interviewee described her experience:

“We have a checklist of anatomical structures that should be identified each day. We always struggle to complete that list, and therefore have no time to review our emotions. One day, I placed my atlas book on the face of my cadaver while dissecting just to see the images more clearly. Suddenly, I realized that I was treating his body like a learning aid. I felt terrible for what I did.”

Another student stated that he tended to dissect mechanically because of the current method of dissecting cadavers:

“In our curriculum, cadaver dissection is proceeded by specific parts or organs. For example, hands today, forearms tomorrow… After focusing on small structures inside a part for three or four hours, I often feel like I am dealing with a part of some machine rather than a human body. It is difficult to think the wholeness of the body with the present way of learning.”

The participants also described their negative emotional experiences during cadaver dissection. Many students mentioned their shock, apprehension, and anxiety when facing cadavers for the first time. Among these remarks, the authors found an interesting emotional response from several students who felt the fear of death during their first encounter. One participant revealed his personal experience:

“The cadaver somehow reminded me of my grandmother who had passed away when I was a small child. As we were really close, I was deeply sorrowful and afraid of the fact that anyone could die. At the funeral, I saw her body in a coffin. I still remember that sight and even the smell of the room. Those memories resurfaced when I saw the cadaver for the first time, making me afraid that death was always beside me.”

Most of the students stated that they easily overcame their fear after a few dissections and became friendly with their cadavers. However, a minority of them expressed intense and prolonged guilt towards the donors that made them uncomfortable in the gross anatomy laboratory. While attempting to explore the reasons for their guilt, the authors identified the following factors; the discomposure of viewing disassembled bodies, a sense of incompetence in dissection skills, and self-blame for insufficient progress through the course. During the interviews, the participants frequently used the expressions that cadavers were “dismembered” or “mutilated”, which reflected their emotional discomfort at viewing disassembled bodies. Also, the students tended to feel that they were not well prepared for human dissection in terms of dissection skills, and felt guilty when their efforts and outcomes seemed to be insufficient in fulfilling donors’ expectations. One interviewee questioned whether she had learned and improved adequately as the donor would have desired:

“I have learned many things in the gross anatomy laboratory, but I am not sure that my learning experiences are worth sacrificing human bodies. In terms of opportunity cost, one body can save many lives if we donate the organs. The donor wanted me to become a great doctor who saves many patients. Yet, I am not confident that I have improved enough despite using this learning opportunity.”

### Emotional impact of memorial ceremonies

Almost all the interviewees agreed that the memorial ceremonies for cadaver donors were impressive and meaningful. They were proud of our tradition of emphasizing the humble and professional attitude of medical students, and expected the ceremonies to continue and increase. The ceremonies positively stimulated students’ emotions of gratitude, respect, and responsibility. One participant described the role of the ceremonies as follows:

“Under the pressure of examinations, I often forget the fact that I am dealing with a real human body, and conduct the dissection mechanically. Fortunately, I have a chance to reconsider and correct my attitude towards the cadavers during the memorial ceremonies. The annual ceremony with the donors’ families was very impressive and I could actually comprehend that the cadavers were once alive and were someone’s beloved family member.”

Besides the major annual events, the daily ceremonies were also found to have educational effects on medical students. The participants emphasized that the signboards on their dissection tables and the daily silent tributes frequently reminded them of their initial emotions towards the donors and therefore helped them in maintaining proper attitudes during the dissection. One interviewee described her experience:

“Since my cadaver is a little bit obese woman, sometimes I have difficulties in identifying target vessels or nerves. One day, I was quite irritated because I spent too much time to remove fat tissues. Honestly, I said some bad words insulting her body during the dissection. Then, I suddenly saw the picture of her smiling face in the signboard and felt really sorry for her. Without that experience, I might not have a chance to correct my fault.”

The other participant mentioned the role of the daily memorial ceremonies in promoting humanistic values among students:

“At first, I thought the daily silent tributes were too formal and superficial events. But, I had changed my perspective after conducting the ceremonies for weeks. During the dissection, I often feel like the cadaver is just a learning aid. Actually, we often call and treat the body as an inanimate object. The daily ceremonies are really helpful in reminding that the cadaver is a human being. I always try to remember it by calling my cadaver’s name during the silent tributes.”

## Discussion

The emotional experiences of students in the gross anatomy laboratory could have significant impacts on their learning outcomes and professional identity formation. The close relationship between learning and emotion has been suggested by numerous educators, since the time of the early Greek philosophers like Aristotle. In this study, we focused on the educational theory of ‘feelings-as-information’ [17]. It conceptualized the role of subjective experiences, such as emotions, metacognitions, and bodily sensations, in judgment [18], which helped us in understanding the influences of the students’ emotional experiences on their knowledge and attitudes.

Medical students in this study revealed their various emotional experiences during cadaver dissection and memorial ceremonies. Most of their emotional responses to dissections, including anxiety and apprehension as well as responsibility and gratitude, were consistent with our expectations. Unexpectedly, the authors discovered that some students experienced intense and prolonged guilt towards the donors. The emotional results of the memorial ceremonies were quite positive as they prevented the decline of the students’ positive emotions such as respect and responsibility while stimulating their motivation and diligence.

The medical students’ emotional experiences at the beginning of the dissection were a mixture of primary emotions such as shock and apprehension and refined emotions like gratitude and responsibility. Most of the negative emotions were instant and reactive, and spontaneously diminished, as previous studies described [19, 20]. However, a minority of the students seemed to feel an intense and sustained repulsion towards dissection because it revived their fundamental fear of death. These students require more individualized approaches and interventions to enable them overcome this fear.

The students possessed compelling senses of purpose and responsibility when they began the dissection course, however, these emotions decreased gradually as the class progressed. In this study, the authors indicated that the excessive workload and organ-based approach during dissection class could make the students rather emotionless. The gross anatomy laboratory can overwhelm medical students with an increasing workload of cadaver dissections, thus numbing their emotions [21, 22]. In addition, the current dissection procedure focusing on specific body parts could hinder medical students from perceiving cadavers as human bodies and treating them with respect. Introducing system-based or problem-based teaching methods in the gross anatomy laboratory can assist in cultivating appropriate attitudes among students [23–25].

Although medical students’ predominant emotional responses to the cadaver donors were gratitude and respect in accordance with previous studies [26–29], the authors discovered underlying guilt towards the donors among a minority of students. While research has reported medical students’ guilt during cadaver dissection [30, 31], our understanding of this emotional response is limited. In this study, several factors were suggested as the reasons for students’ guilt. Previous studies have comprehensively described that medical students might experience shock or disgust due to visual, olfactory, or tactile stimuli from cadavers [10, 32–34]. However, the process of these stimuli engendering guilt seems to be influenced by Confucian tradition in East Asian countries that regards dissection as inhumane and immoral [13, 35]. The other contributor was students’ perception of their incompetence in performing accurate dissections. Also, students tended to believe that in terms of its impact and outcome, body donation for their learning was less valuable than other options like organ donation for transplantation. As the self-degradation among medical students might provoke or reinforce their guilt, anatomy teachers should make educational efforts to let them participate in cadaver dissection with pride and self-confidence.

Based on these results, the authors recommend that educators should emphasize the following subjects during prior education to prepare medical students emotionally for dissections. First, dissection is purposeful behavior far removed from “mutilation”, although severe disassembling of cadavers is required. Second, students’ ineptness is a normal and reasonable phenomenon, in complete accordance with donors’ expectations. Therefore, they need not feel distressed or guilty for their mistakes. Third, the value and effect of the dissection experiences in undergraduate medical education are not visible immediately but, throughout the students’ medical careers they are gradually revealed. Additionally, the authors suggest that anatomy professors should focus on the few students who display prolonged apprehension and aversion to dissection, and consider providing alternative learning opportunities such as prosection-based or computer-based tutorials.

The memorial ceremonies for cadaver donors were effective in preventing the decline of motivation and responsibility among students, as reported in previous studies [36, 37]. The large-scale ceremonies provided students with opportunities to pause during their tasks and assess their emotions, thereby reminding them of the wholeness and dignity of human bodies. The daily rituals, such as reading donors’ will or paying brief tribute, were also powerful in promoting appropriate perspectives and attitudes towards cadaver dissection. These events could make medical students feel their relationships with cadaver donors more friendly, therefore ensure respectful handling of the bodies.

### Study limitations

The present study adopted a qualitative approach in exploring the complex emotional responses of medical students to cadaver dissection and memorial ceremonies. An interviewer with expertise in qualitative assessment conducted in-depth focus group interviews on the large number of students during two consecutive school years. However, this study has some limitations. First, the results only reflect the opinions of medical students from a single institution. Second, the students who volunteered for the study may have more intensive emotions or perceptions regarding this subject. Third, since the data was collected only cross-sectionally, the subjects had to recall their experiences from when they began the course. Fourth, the study is limited to the students’ views and does not include the perspectives of anatomy teachers or donors’ families. Finally, the results have not been legitimized by the study participants. Additional focus group or personal interviews with the students to assess if the results accurately represent their emotions and perceptions could improve the credibility of the study. Moreover, longitudinal studies following the subjects through their school and training years could provide a deeper understanding of the impact of emotional experiences during cadaver dissection on their professional identity formation.

## Conclusions

The gross anatomy laboratory provides intensive emotional experiences for medical students and has a significant influence on their professional identity formation. Educators should endeavor to help students in overcoming their initial distress at facing cadavers and sustaining their sense of responsibility and respect throughout the dissection course. In addition, anatomy teachers need to pay attention to students’ guilt towards cadaver donors that makes them distressed and distracted during dissections. Professors can reduce their guilt and promote positive emotional experiences by teaching that dissection is an interesting and valuable task and they deserve this opportunity. Memorial ceremonies, including not only large-scale events but also daily rituals, play an important educational role in developing appropriate attitudes among medical students in the gross anatomy laboratory. The ethical and humanistic values cultivated by the memorial ceremonies could be an important building block to reinforce students’ professional identity formation as medical professions. Anatomy teachers should help students in fully utilizing these valuable learning opportunities by letting them actively participate in the ceremonies.

## Additional files

Additional file 1: Appendix 1. Gross anatomy laboratory schedule of the Korea University College of Medicine. (DOCX 16 kb)

Additional file 2: Appendix 2. An example of the checklists. (DOCX 17 kb)
