# Supplementary material for: Emotional experiences of medical students during cadaver dissection and the role of memorial ceremonies: a qualitative study
Source: BMC Med Educ. 2018 Nov 12;18:255. doi: 10.1186/s12909-018-1358-0 (PMC6233563; doi:10.1186/s12909-018-1358-0)
Supplement: Supplementary file 1 — Appendix 1. Gross anatomy laboratory schedule of the Korea University College of Medicine. (DOCX 16 kb) [file 12909_2018_1358_MOESM1_ESM.docx]

**Appendix 1** Gross anatomy laboratory schedule of the Korea University College of Medicine

| **Weeks** | **Days** | **Topics** | **Subtopics** |
| --- | --- | --- | --- |
| 1 | 1 | Introduction | |
|  | 2 | Lower limb | Superficial structures of anterior and medial thigh |
|  | 3 |  | Deep structures of anterior and medial thigh & Superficial structures of gluteal region and posterior thigh |
| 2 | 4 |  | Deep structures of gluteal region and posterior thigh Popliteal fossa |
|  | 5 |  | Leg and foot |
|  | 6 |  | Foot and lower limb joints |
|  | 7 | Upper limb | Superficial structures of upper limb and posterior scapular regions |
| 3 | 8 |  | Axilla and arm |
|  | 9 |  | Cubital fossa and forearm |
|  | 10 |  | Hand and upper limb joints |
| 4 | 11 | Examination I | |
|  | 12 | Back | Vertebrae and superficial back |
|  | 13 |  | Muscles and nerves of the back |
| 5 | 14 | Thorax | Anterior thoracic wall and breast |
|  | 15 |  | Mediastinum |
| 6 | 16 |  | Heart |
|  | 17 | Abdomen | Anterior abdominal wall and inguinal canal |
|  | 18 |  | Peritoneum and foregut |
|  | 19 |  | Midgut and hindgut |
|  | 20 |  | Posterior abdominal wall |
| 7 | 21 | Pelvis and Perineum | Pelvic bone and pelvic viscera |
|  | 22 |  | Pelvic viscera |
|  | 23 |  | Pelvic wall, nerve, vessels, perineum |
| 8 | 24 | Examination II | |
|  | 25 | Annual ceremony with the donors’ families (gam-eun-je) | |
| 9 | 26 | Head and Neck | Skull |
|  | 27 |  | Face |
|  | 28 |  | Brain coverings |
| 10 | 29 |  | Orbit |
|  | 30 |  | Ear |
|  | 31 |  | Parotid gland and temporal region |
|  | 32 |  | Nasal cavity |
| 11 | 33 |  | Oral cavity |
|  | 34 |  | Anterior, posterior triangle of neck |
|  | 35 |  | Pharynx, larynx |
| 12 | 36 | Examination III | |
