# Supplementary material for: Emotional experiences of medical students during cadaver dissection and the role of memorial ceremonies: a qualitative study
Source: BMC Med Educ. 2018 Nov 12;18:255. doi: 10.1186/s12909-018-1358-0 (PMC6233563; doi:10.1186/s12909-018-1358-0)
Supplement: Supplementary file 2 — Appendix 2. An example of the checklists. (DOCX 17 kb) [file 12909_2018_1358_MOESM2_ESM.docx]

**Appendix 2** An example of the checklists

| **Dissection team ( ) Name ( )**  **Topic:** Lower limb  **Subtopic:** Superficial structures of anterior and medial thigh  **0 :** Fail to dissect  **1 :** Dissected, but damaged  **2 :** Correctly dissected | | | | | | | |
| --- | --- | --- | --- | --- | --- | --- | --- |
| **Anatomical structures** | **Lt.** | | | **Rt.** | | | **Notes** |
|  | 0 | 1 | 2 | 0 | 1 | 2 |  |
| Superficial inguinal lymph node |  |  |  |  |  |  |  |
| Ant. femoral cutan. nerve |  |  |  |  |  |  |  |
| Lat. femoral cutan. nerve |  |  |  |  |  |  |  |
| Great saphenous vein |  |  |  |  |  |  |  |
| Saphenous opening |  |  |  |  |  |  |  |
| Superficial external pudenal a. |  |  |  |  |  |  |  |
| Superficial epigastric a. |  |  |  |  |  |  |  |
| Superficial external iliac a. |  |  |  |  |  |  |  |
| Femoral nerve |  |  |  |  |  |  |  |
| Femoral vein in femoral sheath |  |  |  |  |  |  |  |
| Lymphatics in femoral canal |  |  |  |  |  |  |  |
| Femoral artery |  |  |  |  |  |  |  |
| Tensor fasciae latae |  |  |  |  |  |  |  |
| Iliacus |  |  |  |  |  |  |  |
| Sartorius |  |  |  |  |  |  |  |
| Adductor longus |  |  |  |  |  |  |  |
| Rectus femoris |  |  |  |  |  |  |  |
| Vastus medialis |  |  |  |  |  |  |  |
| Vastus lateralis |  |  |  |  |  |  |  |
| Patella ligament |  |  |  |  |  |  |  |
| Pectineus |  |  |  |  |  |  |  |
| Profunda femoris a. |  |  |  |  |  |  |  |
| Gracilis |  |  |  |  |  |  |  |
| Iliotibial tract |  |  |  |  |  |  |  |
| Saphenous nerve |  |  |  |  |  |  |  |
